# Computer guided temporomandibular joint reconstruction of Kaban III hemifacial microsomia with anotia: A case report

**DOI:** 10.1016/j.ijscr.2019.03.005

**Published:** 2019-03-16

**Authors:** Mamdouh Ahmed, Sherif Ali

**Affiliations:** a Oral and Maxillofacial Surgery Department, Faculty of Dentistry, Cairo University, Cairo, Egypt; b Cranio-maxillofacial surgery Department, Nasser institute for research and treatment, Cairo, Egypt

**Keywords:** Craniofacial microsomia, Kaban III, Computer-assisted surgery, Reconstructive surgery, Temporomandibular joint

## Abstract

INTRODUCTION, Hemifacial microsomia is the second most common craniofacial congenital anomaly. It corresponds to a group of malformations ranging from minimal facial asymmetry to sever form affecting mandible, soft tissues, orbit, ear, and cranial nerves. PRESENTATION OF CASE, We present a case of 6 years old patient with Kaban class III hemifacial microsomia with anotia. Temporomandibular joint was reconstructed by costochondral graft using computer guided surgery (simulation and rapid prototyping). A computer guided soft tissue guide, mandibular, and maxillomandibular/zygomatic models were constructed using rapid prototyping technology. The customized computer guided soft tissue guide was used to localize the proper position of skin incision, the mandibular model was used for preoperative reconstruction plate bending, and the maxillomandibular/zygomatic model was used to estimate the rib graft length and position. Postoperative assessment showed proper positioning of the graft, with no complications or facial nerve affection. DISCUSSION, In this report, we introduce a new computer guided technique to estimate and identify the proper position of the temporomandibular joint graft based on patient CT. This technique eliminated the need of extended incisions with excessive dissection and provided a more accessible field for rib graft fixation, facilitating the surgical procedures. CONCLUSION, The use of computer guided surgery (simulation and rapid prototyping) for temporomandibular joint reconstruction in Kaban III hemifacial microsomia with anotia facilitates the surgical procedure, minimizes procedure time, increases precision, and reduces possible complications.

## Highlights

• Localization of TMJ graft position represents a true dilemma in Kaban III hemifacial microsomia with anotia.
• Computer guided soft tissue guide eliminates the need of extended skin incisions with excessive dissection.
• Maxillomandibular/zygomatic complex model facilitates the procedures, and allows proper positioning of graft according to the preoperative plan.
• Computer guided surgery facilitates the surgical procedures, reduces operation time, decreases complications, and increases precision.

## 1 Introduction

Hemifacial microsomia (HFM) is the second most common craniofacial congenital anomaly after cleft lip and/or palate. It corresponds to a group of malformations affecting first and second brachial arches structures [1]. Clinical picture of HFM varies from mild form with minimal facial asymmetry and preauricular skin tags to sever form affecting mandible, soft tissues, orbit, ear, and cranial nerves [2]. Different systems have been used in a trial to classify these various forms of deformities. In 1969, Pruzansky introduced his classification based on the degree of mandibular hypoplasia. This classification was modified by Kaban et al in 1988. Kaban classification adds the position of the temporomandibular joint (TMJ) and glenoid fossa to the previous classification. Thereafter, more comprehensive classification as Orbital Mandible Ear Nerve Soft tissue (OMENS), OMENS+, and CFDS have been introduced [[3], [4], [5], [6], [7]].

HFM requires longitudinal, staged, multidisciplinary treatment. Correction of skeletal maxillomandibular deformity represents the corner stone in restoring facial symmetry. This step is very challenging in Kaban III cases. Absence of mandibular ramus and TMJ indicates the need of TMJ reconstruction with bone grafting and/or distraction. Costochondral graft (CCG) represents the gold standard for TMJ reconstruction. However, localization of the forthcoming TMJ position represents a true dilemma in Kaban III cases due to altered position of glenoid fossa associated with the complete absence of TMJ, deficient soft tissues, deformed (or absent) ear, and loss of anatomical landmarks [[8], [9], [10], [11]].

This case report was prepared following the SCARE Guidelines [12]. The purpose of this study was to introduce a new computer guided technique to estimate and identify the proper position of the TMJ graft in Kaban class III HFM with anotia using computer guided soft tissue guide, and 3D printed models.

## 2 Patient information

A 6 years old male patient with HFM referred to our institution for TMJ and mandibular reconstruction. The patient was free from any other medical conditions. Family history revealed that no other family member had similar condition.

## 3 Clinical findings

**Fig. 1:**
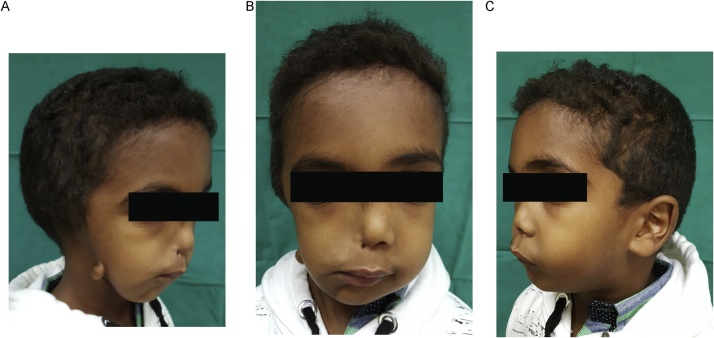
Clinical photos of the patient before the surgery.

Clinical examination showed an underdeveloped right side of the face, severe mandibular deviation toward the right side, deviated right mouth corner, absent ala of the nose, concave lower right facial profile, sever soft tissue dificiency, anotia with preauricular tag, normal orbital size and position for both sides. Palpation of the right side revealed absence of TMJ and ramus. Facial nerve testing showed normal nerve function. Intraoral examination showed normal tongue, with no canting. Aural examination (by ENT specialist) showed right hearing loss, atresia, and anotia (Fig. 1).

## 4 Radiographic assessment

Computed tomography (CT) was performed for the skull using a multi-slice helical CT machine (I-CAT® Precise™ from I-CAT® Technology, Hatfield, PA). Image acquisition was performed with 1 mm slice thickness, 0.625 mm slice increment, 0.3 mm voxel size, 17 X 23 cm extended field of view, 120 KV, 5 mA and 4 m exposure time. CT radiographic examination revealed right side absent TMJ, ramus, coronoid process and condyle as well as hypoplastic mandibular body, malar bone and zygomatic arch. The patient was diagnosed as class III HFM with anotia. (Modifiied OMENS+ : O0, OP0, M3, E4, N0, S3, C0)

## 5 Interventions

### 5.1 Preoperative virtual planning and models fabrication

In this step, the virtual planning was performed, the 3D printed models and computer guided soft tissue guide were constructed using rapid prototyping (RP) technology, and the reconstruction plate was initially bent.

### 5.2 Virtual planning

**Fig. 2:**
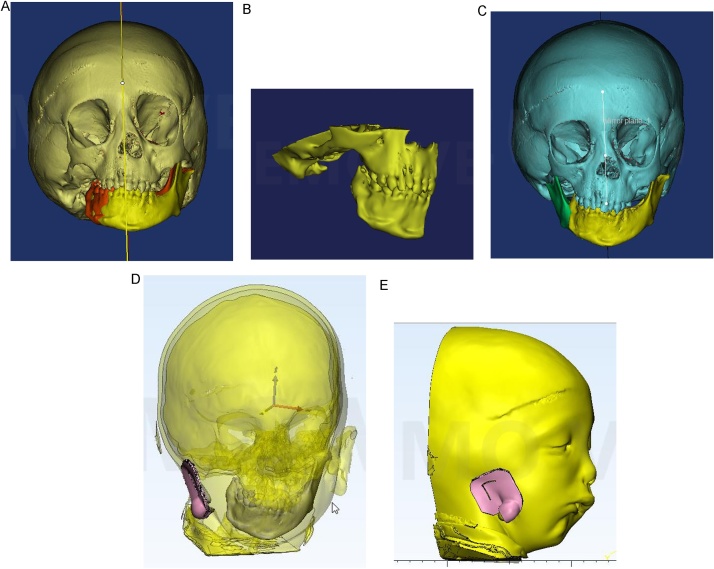
Virtual planning. a) Deviated mandible (red) slightly rotated around the normal TMJ toward the unaffected side (yellow). b) First virtual model (maxillomandibular complex with the zygomatic bone). c) Right mandibular body, ramus, and TMJ (green) virtually reconstructed using mirror imaging of the mandiblar left side. d) 3D soft tissue virtual guide (pink) constructed over the deficient preauricular area with transparent soft tissues to locate the glenoid fossa. e) 3D soft tissue virtual guide (pink) with a slot corresponding to the anticipated soft tissue incision (For interpretation of the references to colour in this figure legend, the reader is referred to the web version of this article).

Computed tomography DICOM (digital Imaging and Communication in Medicine) files were imported to the 3D surgical planning software (Mimics 10.0, Materialise NV, Leuven, Belgium). Through a series of segmentation and simulation processes using the software, the midface and mandible were virtually selected and separated. The deviated mandible was slightly rotated around the normal TMJ toward the unaffected side in the most appropriate occlusion. The maxillomandibular complex with the zygomatic bone was selected to form the first virtual model (virtual 1). Mirror imaging of the mandiblar left side was performed using the facial midline to virtually reconstruct the right mandibular body, ramus and TMJ to generate a mandibular virtual model (virtual 2). Soft tissue thresholding of the DICOM files was performed followed by soft tissue 3D virtual reconstruction of the patient’s head. Using another virtual software (Materialise 3-matic virtual software 11, Materialise NV, Leuven, Belgium), a 3D soft tissue virtual guide was constructed over the deficient preauricular area around the ear tag, with a slot corresponding to the anticipated soft tissue incision guided by the glenoid fossa (Fig. 2).

### 5.3 Rapid prototyping

Stereolithographic (STL) files of soft tissue virtual guide and virtual models were exported to multi‐jet modelling printing machine (InVision Si2, 3D Systems e Rock Hill, SC) and fabricated using plastic material (VisiJet SR 200, 3D Systems e Rock Hill, SC). The fabricated models were used for preoperative reconstruction plate bending and intraoperative adjustment of the rib graft, while the computer guided soft tissue guide was used during surgical procedures.

### 5.4 Plate preparation

**Fig. 3:**
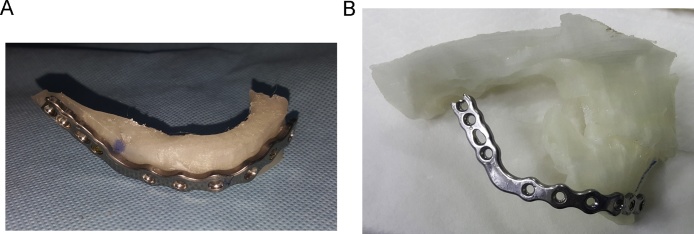
Preoperative plate preparation. a) Reconstruction plate attached to model 2 after preoperative bending. b) Reconstruction plate attached to model 1 to estimate the rib graft length and position.

Mandibular model (model 2) was used for preoperative reconstruction plate bending. The plate was then attached to model 1 to estimate the rib graft length and position (Fig. 3).

### 5.5 Surgical procedures

**Fig. 4:**
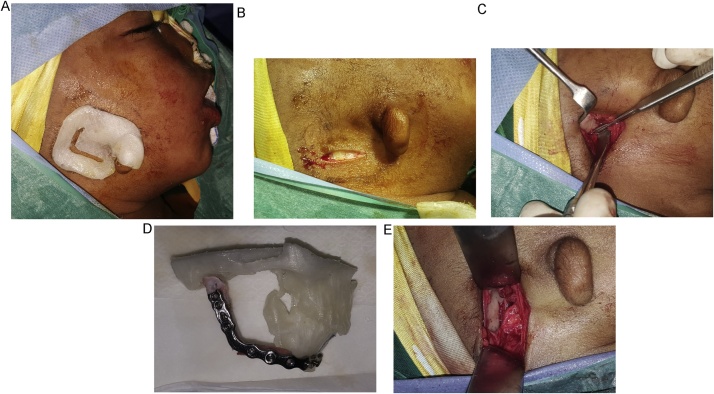
Surgical procedures. a) Soft tissue guide placed in position guided by preauricular skin tag. b) Small skin incision placed over the estimated position of the glenoid fossa. c) Glenoid fossa exposed after minimal blunt dissection. d) Reconstruction plate with the graft attached to model 1. e) TMJ graft in proper relation to the glenoid fossa.

Surgical procedures were performed under general anesthesia using nasotracheal intubation. The soft tissue guide was placed in position on the right side guided by preauricular skin tag and the incision line was marked on the skin. The guide was removed and skin incision was performed. The tissues were bluntly dissected superiorly in the direction of the glenoid fossa. Intraoral vestibular incision was then made in the molar region till the end of the bone stump at the affected side. Finally, a tunnel was formed by blunt dissection between the 2 incisions to create a pocket for graft placement (Fig. 4).

The costochondral graft was harvested from the sixth left rib with the cartilaginous cap based on the preoperative planning, and it was used for reconstruction of TMJ, ramus and body. The reconstruction plate was then attached to model 1, and the rib graft was fixed to the plate in the planed position by the guidance of glenoid fossa of model 1. Reconstruction plate with the rib attached to it was removed from the model and inserted as one unit through the intraoral incision into the prepared pocket. At the end, the reconstruction plate was fixed to the mandible and incisions were closed (Fig. 4).

## 6 Follow up and outcomes

**Fig. 5:**
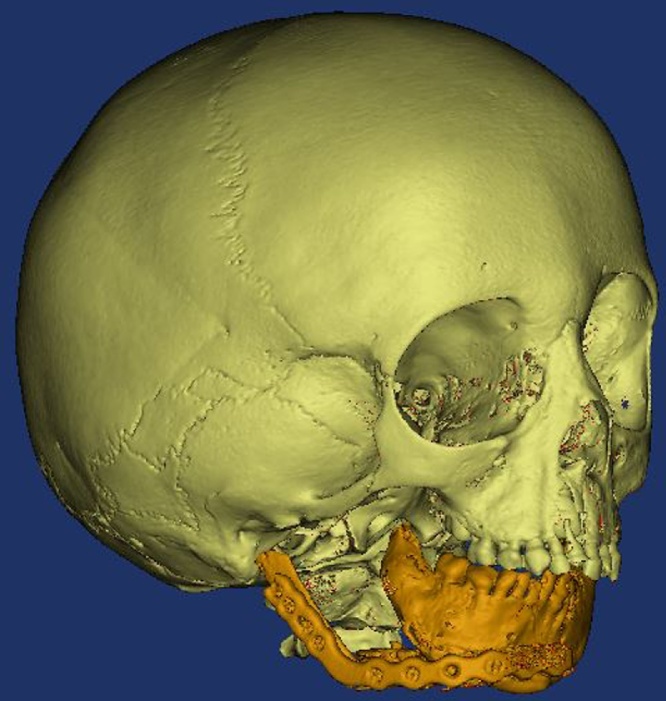
Postoperative CT showing the TMJ graft in proper position according to the preoperative plan.

The patient was instructed to follow soft diet and liquid regime for 1 week, then he was recalled after 1 week for suture removal and clinical assessment. Postoperative CT was performed to confirm proper position of the rib graft. Clinical assessment showed no postoperative complications (dehiscence or infection), and facial nerve testing showed normal facial nerve function. Radiographic assessment showed proper positioning of the graft in relation to the glenoid fossa according to the preoperative plan (Fig. 5).

## 7 Discussion

Correction of skeletal maxillomandibular deformity is the focus of HFM treatment. Different treatment protocols have been introduced. They mainly differ in the technique to be used (grafting, distraction osteogenesis, and/or orthognathic surgery) and the timing of the different procedures. The choice of the proper protocol depends mainly on deformity type and patient’s age; taking family wishes into account [7,8]. In Kaban III HFM with insufficient bone, multi-stage reconstruction is the method of choice. Early reconstruction of TMJ with costochondral graft is the gold standard. This stage can be followed by multi-stage procedures until skeletal maturity [9,13,14].

Computer-guided surgical planning and simulation showed promising results regarding precision and operation time when compared to the traditional methods. Furthermore, it seems to result in better esthetic and functional outcomes. The increased cost of Computer-guided technique may limit its generalization. However, the increased cost due to model constructions can be partially compensated with the reduction in the operation time [[15], [16], [17]]. In this report, we used the 3D RP technology to: (1) accurately localize skin incision; (2) prepare the reconstruction plate; (3) determine the rib graft length; (4) estimate the proposed position of the upcoming TMJ; (5) accurately set the graft in the proposed position.

Preauricular, Risdon and submandibular incisions have been previously used to access TMJ in HFM patients. However, this step presents a true obstacle in Kaban III HFM with anotia [10,14]. In this report, we introduce the use of customized computer guided soft tissue guide based on the patient CT to localize the proper position of skin incision. Virtual planning for soft tissue incision localization in our case showed promising results. It eliminates the need of more extended incisions with excessive dissection, minimizing possible complications [18].

Proper adaptation of the reconstruction plate is a critical factor for the success. This step was previously performed intraoperatively. However with the introduction of 3D printing technology, construction of mandibular 3D model for preoperative plate bending represents an easier and faster method [19,20]. In our study, we constructed a RP model for the maxillomandibular complex (model 1) aside from the reconstructed mandibular model (model 2). This model was used in both preoperative and surgical phases. Preoperatively, the model was used to estimate the length of the rib graft based on the planed position of the upcoming TMJ. While intraoperatively, the model provided a more accessible field to fix rib graft to the plate, facilitating the surgical procedures, and reducing operation time. Moreover, postoperative CT showed precise adjustment of graft position in relation to the glenoid fossa.

## 8 Conclusion

The use of computer guided surgery (simulation and rapid prototyping) for TMJ reconstruction in Kaban III HFM with anotia facilitates the surgical procedure, minimizes procedure time, increases precision and reduces possible complications. Within the limitations of this study we recommend: (1) the use of maxillomandibular/zygomatic model (aside from the mandibular model) in TMJ reconstruction of Kaban III HFM; (2) the use of 3D soft tissue guide in HFM with anotia.

## Conflict of interest statement

Authors have no conflict of interest.

## Funding

This research did not receive any specific grant from funding agencies in the public, commercial, or not-for-profit sectors.

## Ethical approval

Ethical approval was given by “Research Ethics committee, faculty of dentistry, Cairo university”.

## Consent

Written parental informed consent was obtained from the parent (father) for publication of this case report and accompanying images. A copy of the written consent is available for review by the Editor-in-Chief of this journal on request.

## Author contribution

Each undersigned author has made a substantial contribution to the manuscript.

Professor Mamdouh Ahmed was responsible for surgical planning, surgical procedures, and supervision.

Dr. Sherif Ali was responsible for writing the report, and data interpretation.

## Registration of research studies

This is not ‘First in Man’ report.

## Guarantor

Dr. Sherif Ali, Lecturer of oral and maxillofacial surgery, faculty of dentistry, Cairo university.

## Provenance and peer review

Not commissioned externally peer reviewed.
